# Odor familiarity and improvement of olfactory identification test in Chinese population

**DOI:** 10.3389/fpsyg.2023.1278668

**Published:** 2023-10-16

**Authors:** Hao Zhang, Mingyao Wang, Meiyu Qian, Hongquan Wei

**Affiliations:** The First Affiliated Hospital of China Medical University, Shenyang, China

**Keywords:** odor familiarity, olfactory test, modification, distractor, unpleasant odor, correct recognition rates

## Abstract

**Aims:**

This study aimed to design the Chinese Modified Olfactory Identification (CMOI) test based on the Sniffin' Sticks Olfactory Identification (SSOI) test by changing unfamiliar distractors and odors for more familiar ones for the Chinese population.

**Materials and methods:**

We recruited 200 healthy volunteers (103 males and 97 females, aged 18–65 years, mean age 35.04 years, SD 10.96); in a survey, 100 volunteers rated their familiarity with 121 odors, including all the SSOI test odor descriptors and common odors in Chinese daily life. The SSOI test was modified according to the survey results. The other 100 volunteers were tested three times using the SSOI test, the Modified Distractors Olfactory Identification (MDOI) test established by modifying distractors in the SSOI test, and the CMOI test developed by using familiar unpleasant odors to displace the odors with low correct recognition rates in the MDOI test. We compared the test scores of the volunteers during the modification process.

**Results:**

Volunteers were unfamiliar with 31 odor descriptors in the SSOI test; 23 distractors with low familiarity were displaced with more familiar distractors. The three odors with the lowest correct recognition rate in the MDOI test (apple, leather, and pineapple) were displaced with familiar unpleasant odors. The test scores were significantly higher in the CMOI test than in others (*p* < 0.0001); the correct recognition rate in the CMOI test was significantly higher than in the SSOI test (*p* < 0.01).

**Conclusion:**

The test scores in the CMOI test were significantly improved; it prevented choosing wrongly due to unfamiliarity with an odor and its distractors.

## 1. Introduction

Olfaction is an important sense that can regulate emotions, affects cognition and behavior (Liu et al., 2020; Zambom-Ferraresi et al., 2021), and also reminds us of dangers in the environment (Husain et al., 2021). It can be impaired by chronic rhinosinusitis, head trauma, infections, aging, long-term smoking, alcoholism, metabolic diseases, and autoimmune diseases (Topan et al., 2021). Olfactory dysfunction can significantly affect patients' quality of life (Denis et al., 2021), and it is an early marker of neurodegenerative diseases such as Alzheimer's disease and Parkinson's disease (Cha et al., 2021; Wang et al., 2021).

The diagnosis and classification of olfactory dysfunction mainly depends on olfactory psychophysical tests such as the Sniffin' Sticks test (Hummel et al., 1997), the Connecticut Chemosensory Clinical Research Center test (Cain et al., 1988), the University of Pennsylvania Smell Identification test (Doty et al., 1984), and the T&T test (Takagi, 1987). As one of the most widely used olfactory tests (Niklassen et al., 2018), the Sniffin' Sticks test is composed of the olfactory threshold test, the olfactory discrimination test, and the olfactory identification test (SSOI test) (Hummel et al., 1997).

According to the research by Chrea et al. (2004), differences in culture, customs, and other factors in different countries and regions can lead to different people's familiarity with the same smell. When examinees receive olfactory identification tests that are not suitable for their region, they may be unfamiliar with the odor itself or its distractors, which may affect the results of olfactory tests. Therefore, quite a few researchers designed olfactory test methods suitable for local people (Konstantinidis et al., 2008; Jiang et al., 2010; Ogihara et al., 2011; Yücepur et al., 2012; Fenólio et al., 2022). Among them, the modified scheme for the Sniffin' Sticks test is the most common. SSOI test includes 16 odors and a total of 64 odor descriptors (four for each stimulus), of which 48 are distractors (Hummel et al., 1997). Researchers in different countries and regions, including Spain, Malaysia, Congo, and Turkey, have put forward the modified scheme for the Sniffin' Sticks test suitable for local people by changing odors and distractors (Balungwe et al., 2020; Delgado-Losada et al., 2020; Sai-Guan et al., 2020; Demir et al., 2021).

In recent years, some researchers in China also have been committed to putting forward modified schemes of olfactory tests suitable for Chinese people. The Institute of Psychology of the Chinese Academy of Sciences and the Beijing Anzhen Hospital, Affiliated with Capital Medical University, has put forward the modified scheme of olfactory tests suitable for Chinese people, CSIT, and COIT (Feng et al., 2019; Su et al., 2021). Although they both used odors familiar to Chinese people to modify the olfactory identification test, they ignored two problems. First, both schemes just changed some odors but did not modify the distractors of other options. Second, researchers mostly choose pleasant or neutral odors instead of unpleasant odors in most olfactory test modification schemes, including these two modification schemes. The warning function is an important olfactory function, and most of the odors with warning functions have unpleasant smells, such as burnt smell in case of fire and special smells in case of natural gas leakage.

To detect the olfactory function of examinees more comprehensively, it is necessary to introduce some unpleasant odors with warning functions into olfactory tests. In this study, we modified the distractors in an olfactory identification test appropriately, and some unpleasant odors with warning functions were introduced to modify the olfactory identification test. We intended to modify the Sniffin' Sticks test according to the Chinese population and include unpleasant odors due to their importance in daily life.

## 2. Materials and methods

For the development of this report, the STROBE guide for observational studies has been followed.

### 2.1. Participants

We recruited 200 volunteers with normal olfaction from 2021 to 2022. The first 100 volunteers (42 males and 58 females, aged 18–65 years, mean age 35.70 years, SD 10.88) participated in the odor familiarity survey. The remaining 100 volunteers (61 males and 39 females, aged 18–60 years, mean age 34.38 years, SD 11.00) participated in the modification of the olfactory identification test. They reported having no obvious olfactory disorder and no previous history of nasal craniocerebral surgery. Physical examination showed that both nasal cavities and olfactory clefts were unobstructed. Signed informed consent was obtained. All procedures used in this experiment involving human participants were approved by the First Affiliated Hospital of China Medical University Ethics Committee in 2021 and were in accordance with the Declaration of Helsinki.

### 2.2. Odorants preparation

This experiment used three odorants, namely, tetrahydrothiophene, 2-methylpyrazine, and trimethylindole, purchased from Aladdin Company (Shanghai, China). We dissolved 1 g of trimethylindole in 20 ml of corn oil, while tetrahydrothiophene and 2-methylpyrazine are both liquids that can be directly used, and the amounts of tetrahydrothiophene, trimethylindole, and 2-methylpyrazine used in this experiment were 1, 2, and 300 mg, respectively. The three odorants were added to three blank felt-tip pens (Burgart Messtechnik, Wedel, Germany) by pipette, respectively. Regarding the safety of the odorants, first, after adding an appropriate amount of the three odorants, the pen can be used for 6 months without adding odorants again during the period. Then, we found that the damage of these three odorants to the human body can be ignored by animal experiments and literature review (Yu et al., 1996; Li et al., 2010, 2020).

### 2.3. Test procedure

Sniffin' Sticks Olfactory Identification test: This test comprises 16 felt-tip pen-like devices, and during the testing procedure, only one olfactory pen cap can be opened at a time. The pen tip was placed ~2 cm under the middle of the examinee's double nostrils and did not touch the examinee's skin. The time for the examinee to smell each pen shall not exceed 2–3 s, with an interstimulus interval of about 30 s. After presenting a stick, the examinees were provided with four odor descriptors to select the option that could best describe the presented odor. Even if examinees were uncertain about the odor, they were required to use the exclusion method to make a choice. The test was repeated successively until all 16 odors were presented (Hummel et al., 1997).

### 2.4. Study design

#### 2.4.1. Odor familiarity survey

The familiarity of 121 odors was investigated in 100 volunteers. Based on Niklassen et al.'s (2018) study, we developed an odor familiarity questionnaire containing 121 kinds of odors. The questionnaire included basic information about the volunteers: name, gender, age, contact details, and occupation (strict measures were taken to ensure that the privacy and personally identifiable information of volunteers were not exposed during the research process, and the names of volunteers were hidden and replaced by numbers only). According to the familiarity with the 121 common odors provided in the questionnaire, we used a Likert-type scale for the volunteers to score by using an online or paper questionnaire (the content of the online and paper questionnaires were similar). The scale ranged from 1 to 5, in which 1 is not familiar and 5 is highly familiar. If the volunteers scored 4 or 5 on an odor, they were considered “familiar” with the odor. The number of volunteers who were “familiar” with an odor among 100 volunteers was the final familiarity score of the odor.

#### 2.4.2. Modification of the olfactory identification test

(1) Preparation of the olfactory test: First, we did not change the odors in the SSOI test but adjusted the distractors according to the odor familiarity results and randomly displaced the unfamiliar distractors with the distractors with an odor familiarity score higher than 75 (Gudziol and Hummel, 2009), to form the MDOI test. Then, we used tetrahydrothiophene, 2-methylpyrazine, and trimethylindole to simulate natural gas, burnt smell, and fecal odor, respectively, and distractors were randomly assigned to the three odors.

(2) Olfactory identification test modification: First, the SSOI test was conducted on 100 volunteers. Then, the volunteers were tested by MDOI test after 30 min, with the test process unchanged. Finally, because the odors, distractors, and volunteers participating in the other 13 odors' tests remained unchanged, to reduce the olfactory fatigue of volunteers, the volunteers were only tested with three odors of natural gas, burnt smell, and fecal odor to complete the olfactory identification test.

### 2.5. Statistical analysis

**Figure 1:**
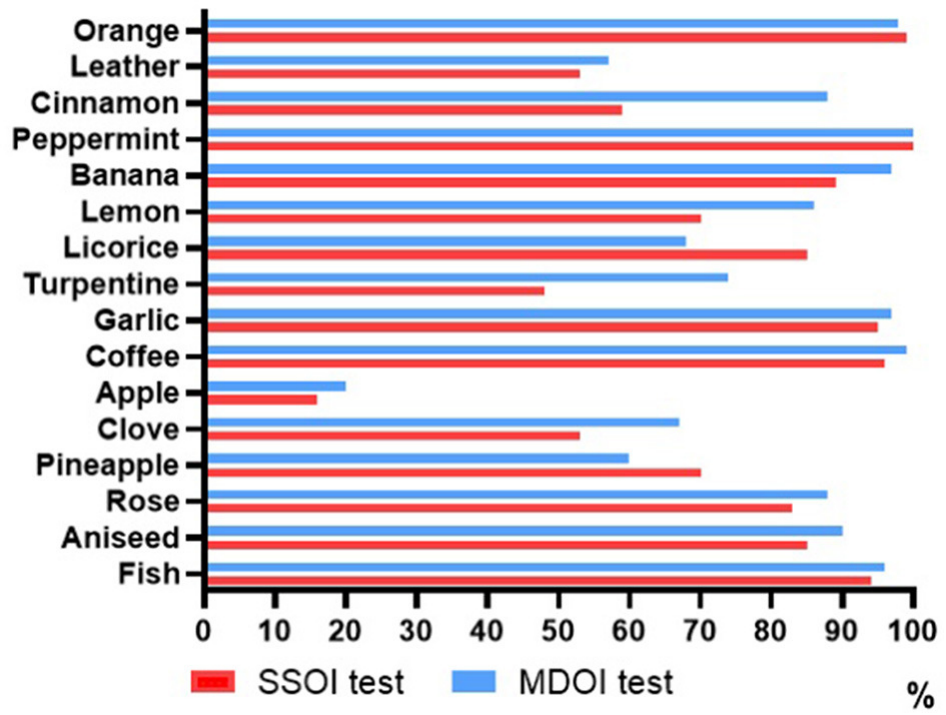
Correct recognition rate of odors in the SSOI and MDOI tests.

**Figure 2:**
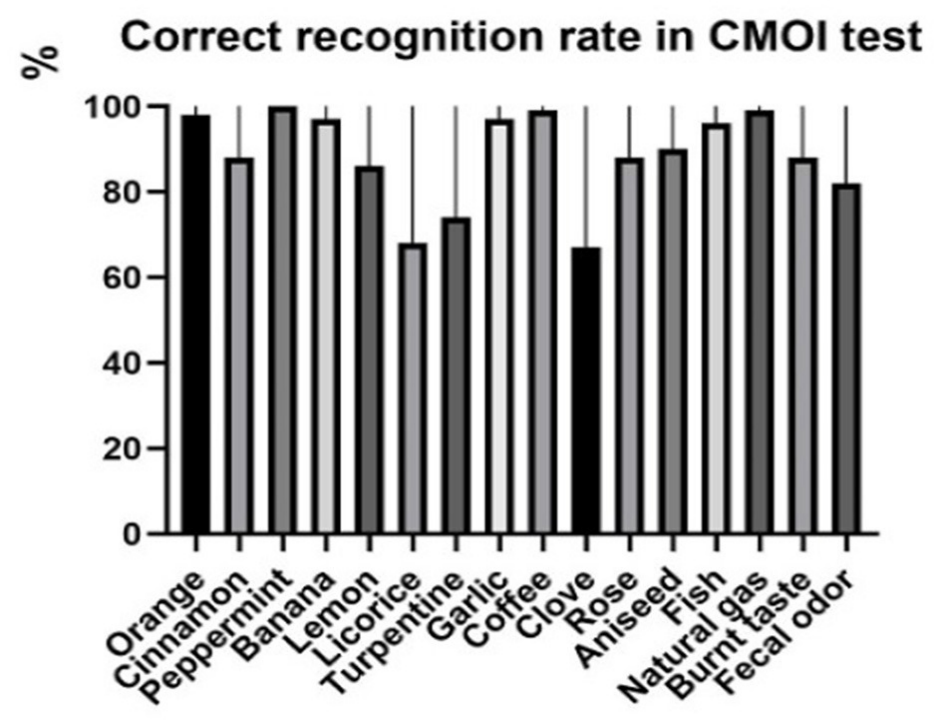
Correct recognition rate of odors in the CMOI test.

R version 3.5.3 was used for statistical analysis. A *p*-value of <0.05 indicated statistical significance. Measurement data are expressed as mean ± standard deviation (x ± s) and counting data as rate (%). The correct recognition rate of odors in each test was calculated, and the test scores in the modification process were tested by paired sample Friedman M-test. The correct recognition rate of odors in the SSOI and CMOI tests was tested by the paired sample Wilcoxon signed rank-sum test. Figures 1, 2 were drawn using the GraphPad Prism 8 (GraphPad Software, La Jolla, CA, USA).

## 3. Results

### 3.1. Odor familiarity test

As shown in Table 1, the most familiar odors in Chinese culture are garlic (95), cantaloupe (93), apple (93), onions (92), orange (91), banana (91), and watermelon (91); the least familiar are fir (6), elderberry (6), raspberry (7), artemisia annua (8), spearmint (10), and oregano (10). Among the 64 odor descriptors in SSOI, 31 odor descriptors had a familiarity of <75 points, such as blackberries (24), fumes (72), glue (70), vanilla (39), fir (6), coconut (59), walnut (73), curry (63), and so on; 23 distractors with odor familiarity >75, including leek (88), paint (88), watermelon (91), mango (90), alcohol (88), chlorine disinfectant (87), pepper (86), and corn (85), were selected to randomly replace the distractors with low familiarity in the first modification.

**Table 1:** Odor familiarity survey results.

**No**.	**Odor**	**%**	**No**.	**Odor**	**%**	**No**.	**Odor**	**%**	**No**.	**Odor**	**%**
1	Apricot	73	32	Pickle	81	63	Spruce	11	94	Cream	70
2	Cranberries	23	33	Coffee	86	64	Coke	38	95	Cabbage	53
3	Peach	90	34	Fumes	72	65	Mint	67	96	Mushroom	74
4	Leather	68	35	Wine	79	66	Pancake	73	97	Green pepper	83
5	Grass	80	36	Rose	72	67	Quill	51	98	Pea	68
6	Cigarette	89	37	Clove	44	68	Strawberry	90	99	Hazelnut	67
7	Glue	70	38	Pepper	86	69	Caramel	57	100	Broccoli	67
8	Vanilla	39	39	Pineapple	89	70	Elder	6	101	Lavender	50
9	Cinnamon	31	40	Plum	49	71	Stables	19	102	Celery	88
10	Honey	76	41	Raspberry	7	72	Paprika	83	103	Balsamic vinegar	82
11	Chocolate	87	42	Rum	11	73	Lox	21	104	Avocado	35
12	Onions	92	43	Fennel	52	74	Orange	91	105	Blackcurrant	26
13	Fir	6	44	Fish	90	75	Timber	77	106	Nutmeg	14
14	Leek	88	45	Bread	85	76	Ginger	84	107	Asparagus	37
15	Watermelon	91	46	Cheese	66	77	Oregano	10	108	Artemisia annua	8
16	Banana	91	47	Salami	27	78	Beetroot	17	109	Truffles	16
17	Cherry	81	48	Ham	68	79	Soybean	67	110	Aniseed	69
18	Walnut	73	49	Pear	83	80	Candy floss	70	111	Eucalyptus	11
19	Coconut	59	50	Cantaloupe	93	81	Peanut	78	112	Green tea	66
20	Grapefruit	53	51	Blackberries	24	82	Butter	51	113	Alcohol	88
21	Apple	93	52	Gasoline	90	83	Fruit sugar	76	114	Chlorine disinfectant	87
22	Lemon	88	53	Curry	63	84	Celery	65	115	Musty smell	73
23	Cookies	61	54	Popcorn	81	85	Corn	85	116	Spoiled oil	42
24	Licorice	31	55	Paint	88	86	Chamomile	24	117	Camphor	53
25	Spearmint	10	56	Bacon	67	87	Tomato	80	118	Sesame oil	81
26	Pine oil	24	57	Almond	66	88	Cucumber	84	119	Natural gas	76
27	Rubber	47	58	Sweat	75	89	Basil leaf	13	120	Burnt taste	84
28	Mustard	74	59	Steak	80	90	Nougat	48	121	Fecal odor	89
29	Thyme	24	60	Soap	84	91	Raisin	74			
30	Carrot	81	61	Ketchup	77	92	Mango	90			
31	Garlic	95	62	Urine	77	93	Seaweed	43			

### 3.2. Establishment of the CMOI test

As shown in Figure 1, the correct recognition rates of leather, cinnamon, lemon, turpentine, apple, clove, and pineapple in the SSOI test were <75%. After the modification of distractors, the correct recognition rate of orange decreased from 99 to 98%. The correct recognition rates of licorice and pineapple also decreased, and that of peppermint was still 100%. The correct recognition rates of all other odors were significantly improved, such as cinnamon from 59 to 88% and lemon from 70 to 86%. However, the recognition rates of apple, leather, and pineapple were still low, which were 20, 57, and 60%, respectively. We added natural gas, burnt smell, and fecal odor into the MDOI test to displace the three odors of apple, leather, and pineapple and established the CMOI test (as shown in Table 2, the bolded words are the correct odors). The results of natural gas, burnt smell, and fecal odor were combined with the test results of 13 odors other than apple, leather, and pineapple in the MDOI test to form the final result of the CMOI test. The correct recognition rate of 16 odors in the CMOI test is shown in Figure 2.

**Table 2:** Final version of the CMOI test.

**Number**	**Descriptor 1**	**Descriptor 2**	**Descriptor 3**	**Descriptor 4**
1	Strawberry	Leek	**Orange**	Pineapple
2	Honey	Mango	**Cinnamon**	Chocolate
3	**Peppermint**	Leek	Onions	Alcohol
4	Chlorine disinfectant	Pepper	Corn	**Banana**
5	Peach	Apple	Burnt paste	**Lemon**
6	Paprika	**Licorice**	Cherry	Ginger
7	**Turpentine**	Fecal odor	Timber	Cucumber
8	Onions	**Garlic**	Sauerkraut	Carrot
9	Cigarette	Wine	Tomato	**Coffee**
10	**Clove**	Pepper	Natural gas	Peanut
11	**Rose**	Cherry	Chamomile	Lemon
12	Green pepper	**Aniseed**	Honey	Paint
13	Bread	Carrot	Gasoline	**Fish**
14	**Natural gas**	Honey	Mango	Chocolate
15	Paprika	Cherry	Ginger	**Burnt taste**
16	Steak	**Fecal odor**	Cucumber	Timber

Bolded words are the correct answers to this test.

Statistical methods were used to analyze the scores of volunteers in the SSOI, MDOI, and CMOI tests. The mean scores in the SSOI, MDOI, and CMOI tests were 11.95 ± 1.37, 12.85 ± 1.37, and 14.17 ± 1.28, respectively. The tenth percentile scores in the SSOI, MDOI, and CMOI tests were 10, 11, and 12.9, respectively. After the paired sample Friedman M-test, the average values of the three groups were not the same (*p* < 0.0001), and the scores of volunteers in the MDOI test were significantly higher than those in the SSOI test (*p* < 0.0001). The scores of volunteers in the CMOI test were significantly higher than those in the MDOI test (*p* < 0.0001) and SSOI test (*p* < 0.0001). The correct recognition rates of odors in the SSOI and CMOI tests were tested by the Wilcoxon signed rank-sum test of paired samples. The correct recognition rates of test odors in the CMOI test were significantly higher than those in the SSOI test (*p* < 0.01).

## 4. Discussion

Based on the SSOI test, the CMOI test significantly improved the olfactory identification test score and the correct recognition rate of odors, by using the distractors and odors more familiar to Chinese people. It may be an effective tool for evaluating the olfactory function of Chinese people.

From the odor familiarity survey, we found that out of the 64 odor descriptors in the SSOI test, the familiarity scores of 31 odor descriptors were <75 points; volunteers were unfamiliar with nearly half of the odors in the SSOI test. When examinees undergo the olfactory test, it is likely that although the examinees perceived the odors, they found it difficult to make a correct choice because they were unfamiliar with the odors or distractors. Comparing the result with the research of Hummel et al. (1997) and Niklassen et al. (2018), we found that Germans were familiar with leather, cinnamon, licorice, rose, clove, etc., and Danish people may be more familiar with leather, vanilla, cinnamon, cheese, bacon, elder, and stables, while Chinese people were more familiar with watermelon, cherry, carrot, peanut, corn, mango, mushroom, and so on, highlighting the significance of modifying the olfactory test.

After adjusting the 23 distractors of the SSOI test according to the odor familiarity survey results, the scores of the olfactory identification test of volunteers were significantly improved. Taking the odor “cinnamon” as an example, in the SSOI test, only 59% of the volunteers could correctly identify the odor “cinnamon”. However, after changing the distractors, the correct recognition rate of the odor “cinnamon” reached 88%. This may be because volunteers were more familiar with the smell of some new distractors and could choose the correct answer through exclusion. However, the correct recognition rates of some odors were still low. The first reason may be that volunteers were unfamiliar with these odors, even if the distractors were modified. The second reason may be that the odor was familiar, but the name was inaccurate. Taking the odor “apple” as an example, there are many varieties of apples in the world, and the smell is not exactly the same, and the apple aroma is relatively light, which can be easily ignored. Therefore, it was difficult for volunteers to make the correct choice in the test. If the names of both odors and distractors are inaccurate, the interference of volunteers may be more serious. The last reason may be the high similarity between odors and distractors, and the difference was insignificant. Taking the odor “apple” as an example again, the distractors of the odor were melon, peach, and orange. In the identification test, most volunteers could only smell fruit flavors but all these four options had fruit flavors, increasing the difficulty of accurate identification. To further improve the score of the olfactory identification test and enable it to have the ability to test unpleasant odors, through a literature search, tetrahydrothiophene, 2-methylpyrazine, and trimethylindole were selected to simulate natural gas, burnt smell, and fecal odor, respectively, and used to displace three odors: apple, leather, and pineapple. After randomly assigning distractors, the CMOI test was composed of 16 odors: 3 new odors and 13 unchanged odors.

In the research studies by Oniz et al. (2013), Kim et al. (2014), and Niklassen et al. (2018), they modified the olfactory identification test by modifying distractors, while in the research studies by Konstantinidis et al. (2008), Oleszkiewicz et al. (2016), and Balungwe et al. (2020), they modified both distractors and odors, and the new odors used by researchers included ginger, honey, eucalyptus, onion, smoked meat, ouzo, Greek grappa, painter oil, wall paint, paint thinner, pepper, and mango. However, no one had proposed using unpleasant odors with warning functions as new test odors.

At present, patients with olfactory disorders mostly use pleasant odor reagents in olfactory training but rarely use unpleasant odor reagents with important warning functions, such as natural gas odorant and simulated burnt odor reagents used in the experiment, which may cause poor recovery of patients' olfactory ability to this kind of odor. In this experiment, the unpleasant smell with a warning function was introduced into the olfactory test, which could better detect the recovery situation of patients' perception of this kind of smell; this was of great significance in evaluating the effect of olfactory treatment. It is hoped to screen patients who cannot correctly identify natural gas, burnt smell, and fecal odor for the next treatment to reduce patients' risk of natural gas leakage, fire, and other dangerous events.

Of course, there are still some limitations to the article. All the volunteers participating in the experiment had normal olfactory senses. Future research should be conducted in patients with olfactory disorders to study how the new CMOI test can distinguish between patients with anosmia and those with hyposmia.

## 5. Conclusion

In this experiment, we modified both distractors and odors in the SSOI test to establish the CMOI test. The odor identification score of healthy volunteers in the CMOI test was significantly improved, reducing instances where volunteers found it difficult to name an odor because they were unfamiliar with the odor and corresponding distractors despite perceiving it. The CMOI test proposed in this study is an effective tool for evaluating the olfactory function of Chinese people.

## Data availability statement

The raw data supporting the conclusions of this article will be made available by the authors, without undue reservation.

## Ethics statement

The studies involving humans were approved by Ethics Committee of the First Affiliated Hospital of China Medical University. The studies were conducted in accordance with the local legislation and institutional requirements. The participants provided their written informed consent to participate in this study.

## Author contributions

HZ: Data curation, Formal analysis, Investigation, Methodology, Writing—original draft, Writing—review and editing. MW: Data curation, Methodology, Resources, Writing—review and editing. MQ: Data curation, Resources, Software, Writing—review and editing. HW: Conceptualization, Funding acquisition, Methodology, Supervision, Writing—review and editing.
